# MicroRNA-27b Depletion Enhances Endotrophic and Intravascular Lipid Accumulation and Induces Adipocyte Hyperplasia in Zebrafish

**DOI:** 10.3390/ijms19010093

**Published:** 2017-12-29

**Authors:** Chia-Chun Hsu, Chi-Yu Lai, Chiu-Ya Lin, Kun-Yun Yeh, Guor Mour Her

**Affiliations:** 1 Department of Radiology, Buddhist Tzu Chi General Hospital, Taichung Branch, Taichung 427, Taiwan; jiajium@tzuchi.com.tw; 2 School of Medicine, Tzu Chi University, Hualien 970, Taiwan; 3 Department of Bioscience and Biotechnology, National Taiwan Ocean University, Keelung 202, Taiwan; c.y.stephen.lai@gmail.com (C.-Y.L.); 10236012@ntou.edu.tw (C.-Y.L.); 4 Division of Hemato-Oncology, Department of Internal Medicine, Chang-Chung Memorial Hospital, Keelung 204, Taiwan

**Keywords:** microRNA sponge, lipogenesis, adipogenesis, liver steatosis, nonalcoholic fatty liver disease

## Abstract

miR-27b has emerged as a regulatory hub in cholesterol and lipid metabolism, and as a potential therapeutic target for treating atherosclerosis and obesity. However, the impact of miR-27b on lipid levels in vivo remains to be determined. Zebrafish lipids are normally stored as triacylglycerols (TGs) and their main storage sites are visceral, intramuscular, and subcutaneous lipid depots, and not blood vessels and liver. In this study, we applied microRNA-sponge (miR-SP) technology and generated zebrafish expressing transgenic miR-27b-SP (C27bSPs), which disrupted endogenous miR-27b activity and induced intravascular lipid accumulation (hyperlipidemia) and the early onset of nonalcoholic fatty liver disease (NAFLD) and nonalcoholic steatohepatitis (NASH). Oil Red O staining predominantly increased in the blood vessels and livers of larvae and juvenile C27bSPs, indicating that miR-27b depletion functionally promoted lipid accumulation. C27bSPs also showed an increased weight gain with larger fat pads, resulting from adipocyte hyperplasia. Molecular analysis revealed that miR-27b depletion increased the expression of genes that are associated with lipogenesis and the endoplasmic reticulum (ER). Moreover, miR-27b-SP increased peroxisome proliferator-activated receptor γ (PPAR-γ), CCAAT enhancer binding protein-α (C/EBP-α, and sterol regulatory element binding transcription factor 1c (SREBP-1c) expression and contributed to lipogenesis and adipogenesis. Conclusion: Our results suggest that miR-27b-SP acts as a lipid enhancer by directly increasing the expression of several lipogenic/adipogenic transcriptional factors, resulting in increased lipogenesis and adipogenesis. In this study, miR-27b expression improved lipid metabolism in C27bSPs, which suggests that miR-27b is an important lipogenic factor in regulating early onset of hyperlipidemia and adipogenesis in zebrafish.

## 1. Introduction

Apart from the classical transcriptional regulators, microRNAs (miRs) have been shown to participate in almost every metabolic homeostasis process, including adipogenesis [1,2], lipogenesis [3], and glucose-stimulated insulin secretion [4,5], in order to exert influence on metabolic pathways which are involved in the pathogenesis of nonalcoholic fatty liver disease (NAFLD) and nonalcoholic steatohepatitis (NASH) [6]. Recent research has demonstrated that miRs are able to regulate the expression of key genes involved in lipid homeostasis, including miR-122, miR-33, miR-106, miR-758, miR-26, miR-370, miR-378, let-7, miR-27, miR-143, miR-34a, and miR-335 [7,8,9,10,11,12,13]. Several miRs have been shown to regulate lipid metabolism-related genes that may be involved in the pathogenesis of NAFLD [6,14,15,16,17]. Therefore, it is plausible that certain miRs participate in the pathogenesis of metabolic diseases.

Karbiener et al., demonstrated that miR-27b levels decreased during adipogenesis of human adipose-derived stem (hMADS) cells and the overexpression of miR-27b repressed PPAR-γ and CCAAT enhancer binding protein-α (C/EBP-α) expression during early onset of adipogenesis and triglyceride accumulation at later stages [18,19]. PPAR-γ has a highly conserved predicted binding site in its 3′ UTR and was confirmed to be a direct target of miR-27b [18]. Kang et al., found that miR-27 is an anti-adipogenic microRNA in part by targeting prohibitin (PHB) and impairing mitochondrial function [20]. In their study, Kang et al., determined that the levels of both miR-27a and miR-27b were down-regulated following adipogenic induction of hMADS, while the overexpression of miR-27a or miR-27b inhibited PHB expression and adipocyte differentiation [20]. In addition, Kong et al., demonstrated that miR-27b plays a central role in the pathogenesis of glucocorticoid (GC)-induced central fat accumulation [21]. Overexpression of miR-27b inhibited brown adipose differentiation and the energy expenditure of primary adipocytes. Conversely, suppressing miR-27b function down-regulated expression of specific genes in brown adipose tissue [21]. Furthermore, Vickers et al., provided in silico, in vitro and in vivo evidence that miR-27b is a strong candidate to be a regulatory hub in lipid metabolism [22]. They demonstrated that hepatic miR-27b is responsive to lipid levels and regulates the expression (mRNA and protein) of key metabolic genes, including angiopoietin-like 3 (ANGPTL3) and glycerol-3-phosphate acyltransferase 1 (GPAM), which have been previously implicated in the pathobiology of lipid-related disorders [22]. Despite these observations, to date, no reports are available regarding the impact of miR-27b on lipid metabolism in vivo.

Numerous studies have documented homologies between zebrafish and human lipid metabolism [23,24,25,26,27]. In fact, the zebrafish has recently become an important model organism for studying NAFLD [28]. Our previous studies have demonstrated that Hepatitis B virus X proteins [29], gankyrin [30], Yin Yang 1 (YY1) [31], and cannabinoid receptor 1 (CB1R) [32] induce the development of hepatic steatosis by increasing the transcriptional activity of lipogenic gene expression in adult zebrafish. Molecular analysis revealed that gankyrin overexpression induced hepatic steatosis and modulated the expression profiles of four hepatic microRNAs, miR-16, miR-27b, miR-122, and miR-126 [30]. In this study, we report the association of YY1 with hepatic lipid accumulation by down-regulating C/EBP homologous protein 10 (CHOP-10) which repressed the expression of PPAR-γ and C/EBP-α in the lipogenic program in zebrafish larvae and adults [31].

The aim of the present study was to explore the effects of depletion of miR-27b on the lipid metabolism of both larval and adult zebrafish. We generated microRNA-sponge (miR-SP) transgenic zebrafish (C27bSP) to analyze the effects of miR-27b depletion on lipid homeostasis. In this study, we characterized the role of miR-27b in lipid metabolism in 27bSP and wild-type zebrafish. We determined the effect of miR-27b depletion on the regulation of pathways that govern lipogenesis and adipogenesis in zebrafish. Excessive inhibition of miR-27b expression is associated with hyperlipidemia, NAFLD (or NASH), and obesity phenotypes. The results from this work reveal, for the first time, that miR-27b functions to regulate molecular pathologic signaling pathways that are involved in lipometabolic disorders in zebrafish.

## 2. Results

### 2.1. Designing and Testing the Functionality of the miR-27b Sponge

**Figure 1:**
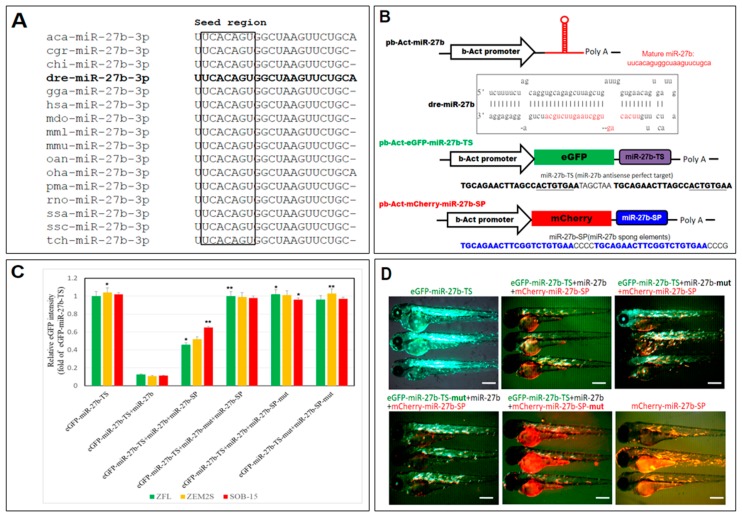
Design and validation of a miR-27b-sponge (miR27b-SP). (**A**) Alignment of the mature miR-27b sequence is perfectly conserved across many species, including arboreal lizard (*Anolis carolinensis*; aca-miR-27b), chinese hamster (*Cricetulus griseus*; cgr-miR-27b), domestic goat (*Capra hircus*; chi-miR-27b), zebrafish (*Danio rerio*; dre-miR-27b), red junglefowl (*Gallus gallus*; gga-miR-27b), humans (*Homo sapiens*; hsa-miR-27b), gray short-tailed opossum (*Monodelphis domestica*; mdo-miR-27b), rhesus macaque (*Macaca mulatta*; mml-miR-27b), mouse (*Mus musculus*; mmu-miR-27b), platypus (*Ornithorhynchus anatinus*; oan-miR-27b), king cobra (*Ophiophagus hannah*; oha-miR-27b), sea lamprey (*Petromyzon marinus*; pma-miR-27b), rat (*Rattus norvegicus*; rno-miR-27b), Atlantic salmon (*Salmo salar*; ssa-miR-27b), wild boar (*Sus scrofa*; ssc-miR-27b) and chinese tree shrew (*Tupaia chinensis*; tch-miR-27b). (**B**) Cloning of pri-miR-27b and miR27b-SP into b-Act expression vectors. Stem-loop structure of premiR-27b is shown, in which mature miR-27b is highlighted in red. (**C**) In vitro EGFP reporter assays were performed to confirm the direct interaction between miR-27b and the target sequences. ZFL, ZEM2S, and SOB-15 cells were transfected with indicated b-Act-miR-27b plasmids, and the EGFP intensity was measured. * *p* < 0.01, and ** *p* < 0.005. (**D**) In vivo EGFP reporter assays were performed to confirm the direct interaction between miR-27b and the target sequences in six days post fertilization (dpf) zebrafish larvae.

The mature miR-27b sequence is perfectly conserved across many species with an identical seed sequence at the 5′ end (Figure 1A). To study the function of endogenous miR-27b in the zebrafish, we designed a miR-27b-sponge containing 10 copies of the miR-27b binding sites and four nucleotide spacers with mismatches opposite miRNA nucleotides 9–12 to block the inhibitory activity of miR-27b (Figure 1B). To evaluate the suppressive activity of the sponge construct in vivo and in vitro, we evaluated whether the sequestration of miR-27b by the sponge products could disrupt the binding of miRNA-27b to target sites in the 3′ UTR of a target mRNA. Thus, we performed an eGFP reporter assay using the luciferase reporter pb-Act-eGFP-mir-27b-TS and a vector, with the 3′ UTR of eGFP mRNA containing a perfect target sequence for miR-27b.

Expression of miR-27b cluster sponge elements (miR-27b-SP) was examined to evaluate its ability to function in vivo to reduce miR-27b expression. Both in vivo and in vitro eGFP reporter assays were performed to confirm the direct interaction of miR-27b-SP and the miR-27b targeting sequence (miR-27b-TS). miR-27b-SP overexpression rescued the reduced GFP intensity of the miR-27b-TS in a consistent manner (Figure 1C) when compared with control GFPs (TS-mut or SP-mut) in in vitro assays. Correspondingly, an in vivo assay demonstrated that the decreased eGFP fluorescence of pb-Act-EGFP-miR-27b-TS/miR-27b co-expression could be rescued by miR-27b-SP expression in dose-dependent manner as compared with the control group (Figure 1D). Collectively, these data suggest that miR-27b-SP can specifically inhibit miR-27b expression and sequester miR-27b activity on its target genes by eliminating miR-27b expression.

### 2.2. Generation of Transgenic C27bSPs (bC27bSP1, 2 and hC27bSP1, 2) Zebrafish Lines

**Figure 2:**
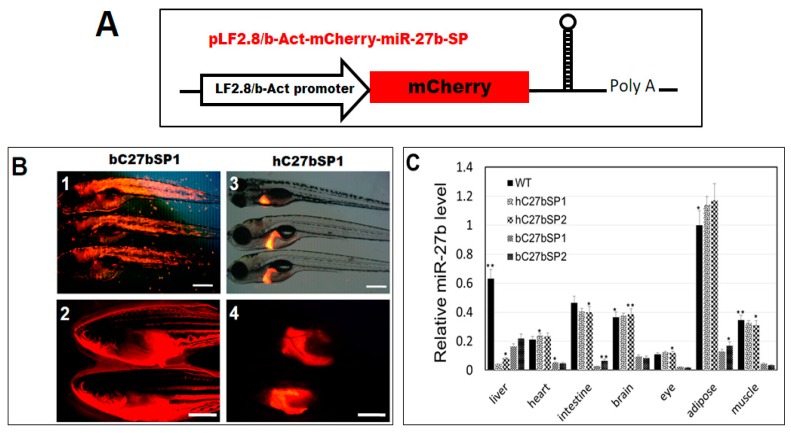
Generation of miR27b-SP transgenic zebrafish. (**A**) Cloning of pri-miR-27b and miR27b-SP into LF2.8 or b-Act expression vectors. (**B**) Red fluorescent images of miR27b-SP expression in the entire body of bC27bSP1 (panel 1, 9 dpf, 40× magnification, scale bars: 200 µm; panel 2, 4 months post fertilization (mpf), 40× magnification, scale bars: 100 mm) and the livers of hC27bSP1 (3, 9 dpf; 4, 4 mpf, 40× magnification, scale bars: 100 mm). (**C**) Stem-loop RT-qPCR analysis of mature miR-27b of the liver, heart, intestine, brain, eye, adipose and muscle tissues from the four transgenic lines, bC7aSPs (bC27bSP1,2) and hC7aSPs (hC27bSP1,2). *n* = 5–8 for each groups. * *p* < 0.01, and ** *p* < 0.005.

To generate stable mCherry-fused miR-27b-SP expression in zebrafish, the pb-Act-mCherry-miR-27b-SP and LF2.8-mCherry-miR-27b-SP constructs were used to produce germline-transmitting transgenic zebrafish lines, C27bSPs (Figure 2A). With the pb-Act-mCherry-miR-27b-SP construct, zebrafish transgenic lines, bC27bSPs (Tg(-2.5β-Act:mCherry-miR-27b-SP)), were generated in which miR-27b expression was globally eliminated (Figure 2B, panels 1, 2). With the *LF2.8-mCherry-miR-27b-SP* construct, zebrafish transgenic lines, hC27bSPs (Tg(-2.8fabp10a:mCherry-miR-27b-SP)), were generated, in which miR-27b expression was specifically eliminated in the liver (Figure 2B, panels 3, 4).

We performed stem-loop RT-PCR to detect expression levels of mature miR-27b in bC27bSPs and hC27bSPs. Two bC27bSPs (bC27bSP1 and bC27bSP2) and two hC27bSPs (hC27bSP1 and hC27bSP2) transgenic lines were selected based on miR-27b expression (Figure 2C). Relative to the wild-type control, miR-27b was significantly down-regulated 15.4- and 7.8-fold in the livers of hC27bSP1 and hC27bSP2 lines, respectively. We additionally demonstrated that the hepatic overexpression of miR-27b-SP did not alter the level of endogenous mature miR-27b in other tissues in hC27bSPs when compared with the wild-type (WT) (Figure 2C). There was a nearly 3.8- and 2.9-fold reduction in hepatic mature miR-27b in the livers of bC27bSP1 and bC27bSP2 lines, respectively. However, there was a significant reduction in the mature miR-27b level in other tissues in bC27bSPs compared with WT (Figure 2C). The results demonstrate that the miR-27b-SP is able to block miR-27b expression in vivo.

### 2.3. Inhibition of Endogenous miR-27b Increases Endotrophic and Intravascular Lipid Accumulation

**Figure 3:**
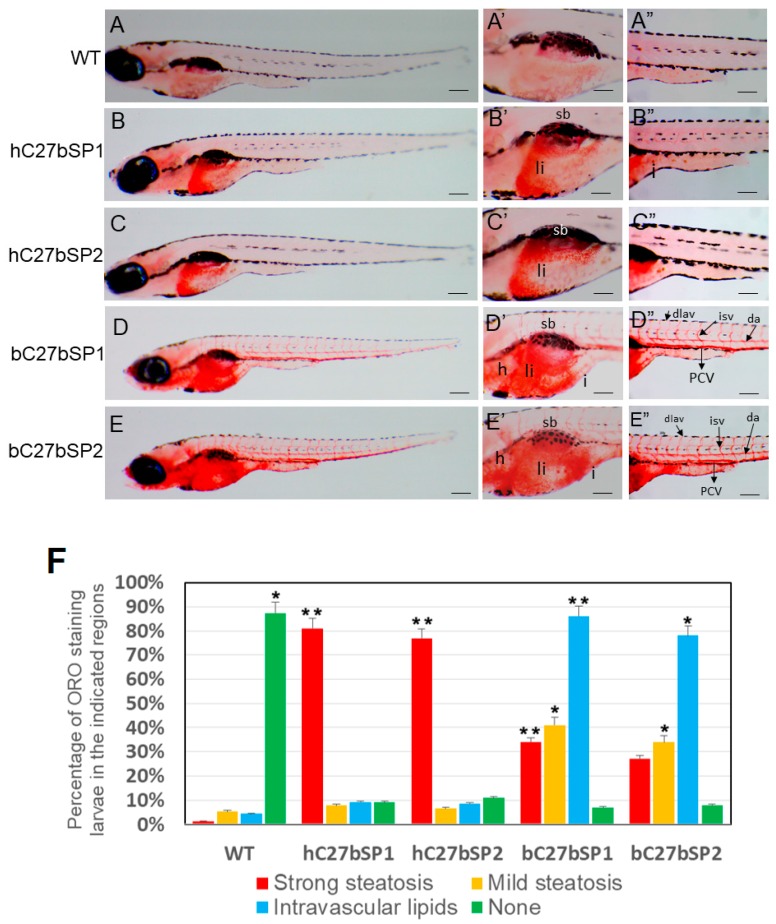
Effect of miR27b-SP on hC27bSPs and bC7aSPs liver and intravascular lipids through Oil Red O (ORO) staining. (**A**–**E**) Lateral view of the whole-mount ORO staining of representative larvae fed with high-fat diet (HFD) (40× magnification, scale bars: 200 µm). Enlargement at the trunk and posterior level is shown in panels ′ and ″ (110× magnification, scale bars: 100 µm). (**F**) The percentages of hC27bSPs and bC27bSPs larvae with steatosis and intravascular lipids was calculated through ORO staining. The ORO staining was performed in triplicate with on average 60–80 larvae per groups. The asterisk represents statistically significant differences; * *p* < 0.01, and ** *p* < 0.005. Abbreviations: e, eye; da, dorsal aorta; dlav, dorsal longitudinal anastomotic vessel; h, heart; i, intestine; isv, intersegmental vessel; PCV, posterior cardinal vein; sb, swim bladder; and ys, yolk sac.

To examine neutral lipids among hC27bSPs, bC27bSPs and WT larvae, 10 days post fertilization (dpf) larvae were stained with Oil Red O (Figure 3). The Oil Red O signal was not detected in WT larvae (Figure 3A,A′,A′′), though it did stain the swim bladder nonspecifically (Figure 3A′). The hC27bSPs larvae showed strong staining only in the liver (Figure 3B,B′,C,C′). The bC27bSPs larvae fed a high-fat diet (HFD)showed moderate staining in the liver (Figure 3D′,E′), brain, and heart (Figure 3D′,E′), with additional staining in the vasculature, including the posterior cardinal vein, dorsal aorta, and intersegmental vessels (Figure 3D″,E″).

Importantly, the incidence of endotrophic and intravascular lipid accumulation (78−86%) and liver lipid accumulation was much higher in hC27bSPs (77−81%) when compared with only 1.4−5.3% liver lipid accumulation and 4.4% intravascular lipid accumulation in WT larvae (Figure 3F). These data suggest that miR-27b depletion can induce endotrophic and intravascular lipid accumulation in zebrafish larvae.

### 2.4. miR-27b Depletion Increases Expression of Genes Associated with Lipogenesis in C27bSPs

**Figure 4:**
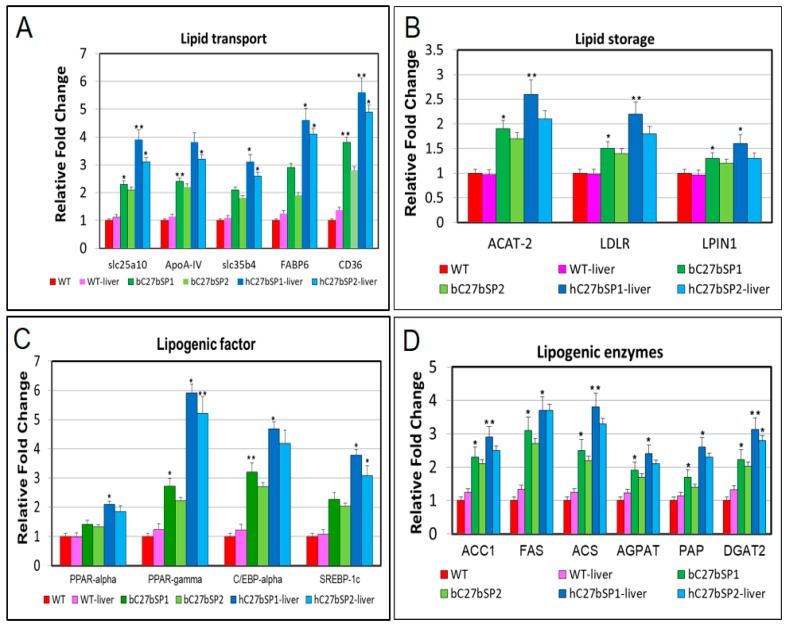
RT-qPCR analysis of selected lipogenic genes in bC27bSPs larvae and livers of hC27bSPs larvae. The relative mRNA expression of lipid transport (**A)** lipid storage (**B**), lipogenic factor (**C**), and lipogenic enzyme (**D**) genes in C27bSPs compared with gene expression in WT fish. The qRT-PCRs were performed in triplicate. Expression analysis of the selected genes using cDNA prepared from average four months male–female fish pairs per groups. Values were normalized against β-actin. The asterisk represents statistically significant differences; * *p* < 0 .01, and ** *p* < 0.005 levels as compared to control.

Because miR-27b depletion can induce endotrophic and intravascular lipid accumulation and hepatic steatosis in zebrafish, we next investigated the effect of miR-27b depletion on the expression of lipogenic and unfolded protein response (UPR) target genes. The mRNA levels of fatty acid (FA) membrane transporters exhibited significant upregulation, including fatty acid transport proteins (FATPs), such as *slc25a10* and *slc35b4*, fatty acid binding proteins (FABPs), such as *FABP6* and fatty acid translocase *FAT*/*CD36* in bC27bSPs and hC27bSPs when compared with WT (Figure 4A). The mRNA levels of lipid storage genes were increased, including *ACAT-2*, the LDL receptor *LDLR*, and *LPIN1* (Figure 4B). Many genes that are involved in hepatic lipogenesis are transcriptionally regulated by sterol regulatory element binding transcription factor 1c (SREBP-1c), including peroxisome proliferator activated receptor-γ (*PPAR*-γ) and CCAAT enhancer binding protein-α (*C/EBP-α*), which were found to be upregulated (Figure 4C). The mRNA levels of key lipogenic enzymes that are involved in fatty acid synthesis were significantly increased, including acetyl-CoA carboxylase 1 (*ACC1*), fatty acid synthase (*FAS*), acyl-CoA synthase (*ACS*), acyl-CoA:1-acylglycerol-sn-3-phosphate acyltransferase (*AGAPT*), phosphatidic acid phosphatase (*PAP*), and diacylglycerol O-acyltransferase 2 (*DGAT2*) (Figure 4D). All of the markers were significantly and highly upregulated in bC27bSPs and in the liver of hC27bSPs when compared with the controls (WT and WT-liver). These results suggest that lipid accumulation in the bC27bSPs and hC27bSPs results from upregulation of genes involved in lipid biogenesis.

### 2.5. hC27bSP Adults Developed NAFLD and Eventually NASH

**Figure 5:**
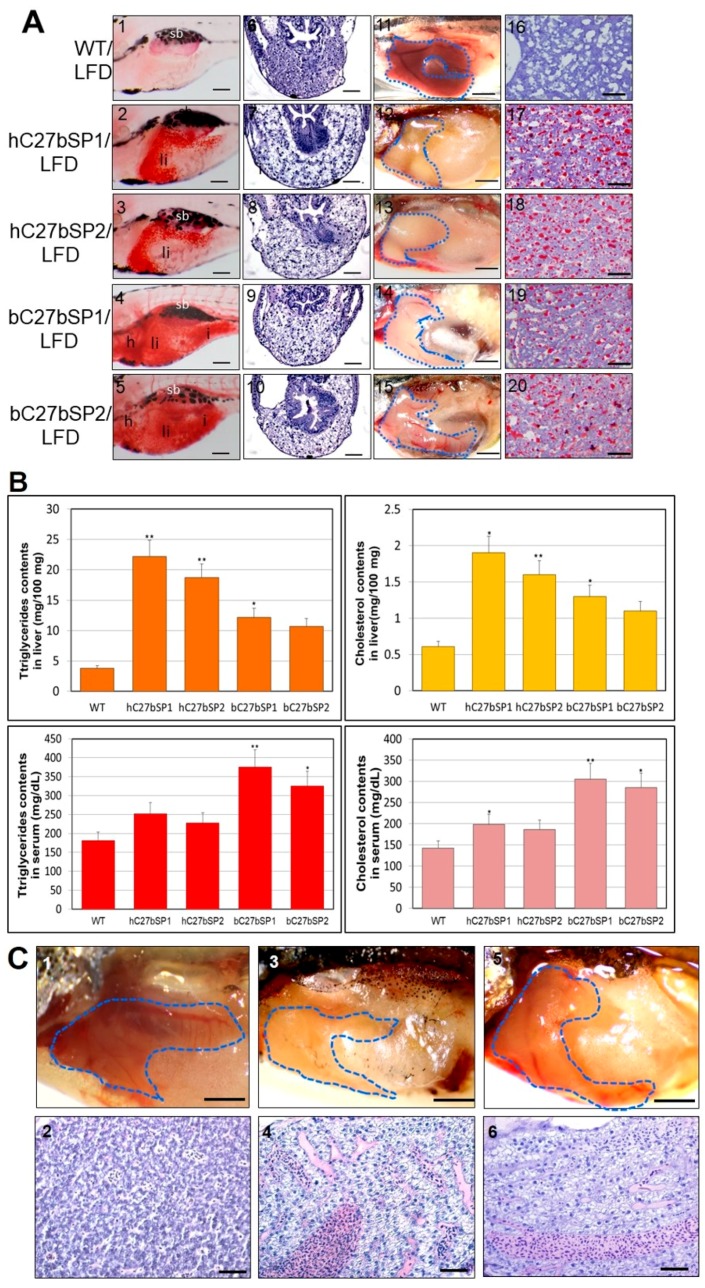

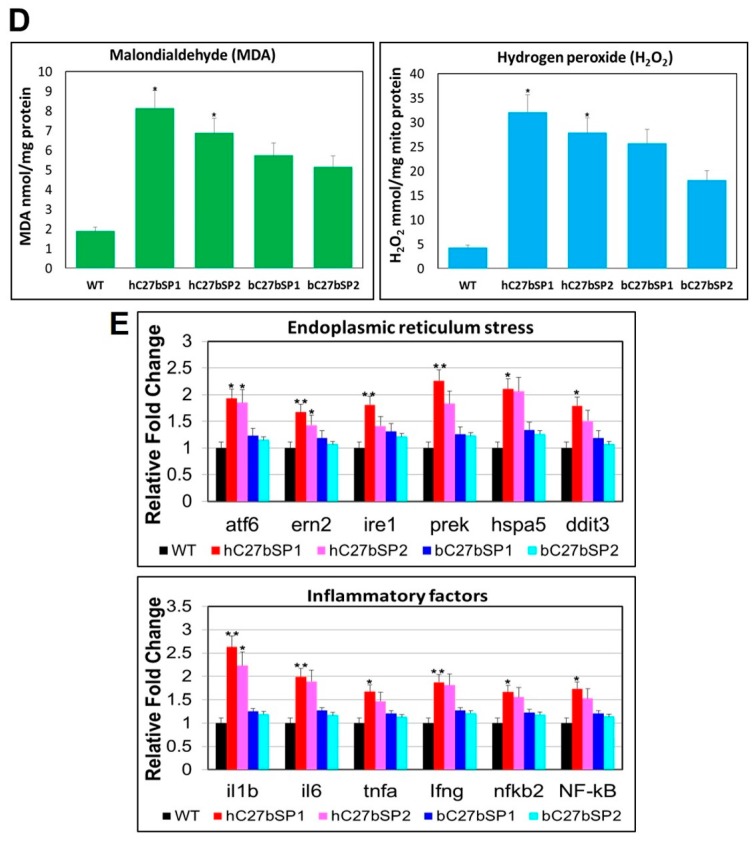
NAFLD and NASH phenotypes observed in hC27bSPs and bC27bSPs adults. (**A**) Liver histological changes of C27bSPs larvae at different quantities of feeding and lipid contents. The ORO staining in the liver of C27bSPs larvae fed with low-fat diet (LFD) for WT (panel 1), hC7aSPs (panels 2 and 3) and bC7aSPs (panels 4 and 5). 110X magnification, scale bars: 100 µm. The histological changes in the liver of zebrafish larvae fed with LFD for WT (panel 6), hC7aSPs (panels 7 and 8), and bC7aSPs (panels 9 and 10), 400× magnification, scale bars: 10 µm. Representative gross anatomy of the liver of 4-month-old WT (panel 11), hC7aSPs (panels 12 and 13) and bC7aSPs (panels 14 and 15) adults. 40× magnification, scale bars: 50 mm. Lipid accumulation was confirmed through ORO-stained sections from adult WT (panel 16), hC7aSPs (panels 17 and 18) and bC7aSPs (panels 19 and 20). 400× magnification, scale bars: 25 µm. (**B**) Comparison of lipid contents (liver and serum triglycerides and cholesterol) in the 11-month-old hC27bSPs and bC27bSPs adults. (*n* = 4–6 for each groups). * *p* < 0.01, and ** *p* < 0.005. (**C**) Representative gross anatomy images of the 11-month-old WT (panel 1), hC27bSP1 (panel 3) and hC27bSP2 (panel 5) livers. 40× magnification, scale bars: 50 mm. H&E staining of the 11-month-old WT (panel 2), hC7aSP1 (panel 4) and hC7aSP2 (panel 6) livers, demonstrating induced liver stress and damage in hC27bSPs compared with WT at the same stage. 400× magnification, scale bars: 50 µm. (**D**) Levels of hepatic MDA, and H_2_O_2_ in hepatic mitochondria was compared with hC27bSPs, bC27bSPs, and WT adults fed a LFD for four weeks. (**E**) Expression of endoplasmic reticulum (ER) stress markers, atf6, *ern2*, *ire1*, *prek*, *hspa5*, *grp78*, and *ddit3* (up) and inflammatory genes, *il-1b*, *il-6*, *tnf-α*, *ifn-γ*, *nfkb2*, and *NF-kB*, is increased (down). The qRT-PCRs were performed in triplicate. Expression analysis of the selected genes using cDNA prepared from average four months male–female fish pairs per groups. Values were normalized against β-actin. The asterisk represents statistically significant differences; * *p* < 0 .01, and ** *p* < 0.005 levels as compared to control.

We suspected the development of early onset of NAFLD phenotypes in the C27bSPs juveniles (<60 dpf). As expected, the C27bSPs juveniles (21 dpf) fed a low-fat diet (LFD) developed obvious liver steatosis, whereas WT juveniles that were fed the same diet exhibited no lipid deposits (Figure 5A, panels 1−5). Histological analysis revealed that homogeneous liver sinusoids and hepatic cells exhibited well-preserved cytoplasm and prominent nuclei in WT (Figure 5A, panel 6) and liver sections of juvenile C27bSPs exhibited hepatic cells with various grades of vacuolization in the cytoplasm (Figure 5A, panels 7−10). Macroscopically, WT adult livers were normal in color, soft, and sanguine at 4.5 months post fertilization (mpf) (Figure 5A, panel 11), whereas approximately 80% of the 4.5 mpf C27bSPs fish showed a yellow and greasy liver, which is typical for a zebrafish fatty liver (Figure 5A, panels 12−15). The fatty liver results were further confirmed using Oil Red O staining, which clearly revealed vesicular steatosis and massive lipid droplets in the liver tissues from adult C27bSPs (Figure 5A, panels 17−20) when compared with those in adult WT (Figure 5A, panel 16). Additionally, we observed a significant increase in the hepatic lipids (5.8-, 4.9-, 3.2-, and 2.8-fold in the hC27bSP1, hC27bSP2, bC27bSP1, and bC27bSP2, respectively, as compared to WT) and cholesterol (3.1-, 2.6-, 2.1-, and 1.8-fold in the hC27bSP1, hC27bSP2, bC27bSP1, and bC27bSP2, respectively, when compared to WT) (Figure 5B). However, the serum lipid and cholesterol levels were only increased in bC27bSPs as compared to hC27bSPs and WT (Figure 5B).

Chronic lipid accumulation within the hepatocytes initiated further hepatocyte damage and liver stress in hC27bSPs adults (older than 10 mpf), which is consistent with previous observations of NASH-like phenotypes (Figure 5C, panels 3−6) and compared with those in WT (Figure 5C, panels 1,2). We discovered that oxidative stress significantly increased the quantity of hepatic MDA and the release of H_2_O_2_ in hC27bSPs and bC27bSPs; however, no or less effect of oxidative stress was observed in the liver of WT that was fed with the LFD (Figure 5D), suggesting that miR-27b depletion can significantly enhance oxidative stress in zebrafish livers. In addition, Molecular analysis of hC27bSP2 NASH livers revealed the upregulation of genes that are involved in NASH development. As expected, hC27bSPs adults exhibited an increased expression of the inflammatory genes *il-1b*, *il-6*, *tnf-α*, *ifn-γ*, *nfkb2*, and *nf-kb* when compared with WT (Figure 5E). In addition, hC7aSPs adults exhibited upregulation of endoplasmic reticulum (ER) stress markers *atf6*, *ern2*, *ire1*, *prek*, *hspa5* and *ddit3* (Figure 5E). These results indicate that hC27bSPs adults developed NAFLD and NASH resulting from the accumulation of fat in the liver, which was accompanied by subsequent activation of inflammatory and ER stress pathways.

### 2.6. miR-27b Depletion Induces Early Onset of Adipocyte Hyperplasia in Zebrafish

**Figure 6:**
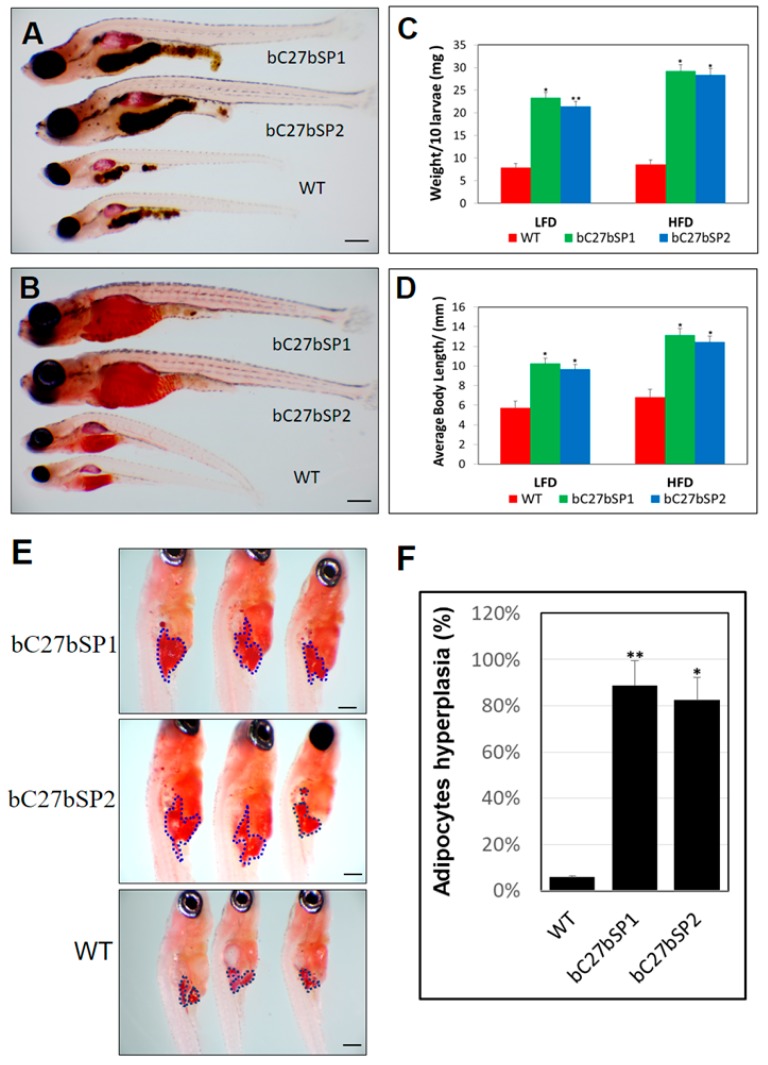
miR-27b depletion results in enhancement of bC27bSPs larval growth and adipocyte formation. (**A**) Representative images of bC7aSPs and WT larvae fed a HFD and (**B**) fed a LFD, 40× magnification, scale bars: 200 µm. (**C**) Average body weights of bC27bSPs and WT larvae fed a LFD or HFD (*n* = 40–50 for each groups). * *p* < 0.01, and ** *p* < 0.005. (**D**) Average body lengths of bC27bSPs and WT larvae fed a LFD or HFD (*n* = 40–50 for each groups). * *p* < 0.01, and ** *p* < 0.005. (**E**) ORO-stained visceral adipocytes in bC27bSPs when compared to WT controls at 24 dpf. Visceral adipocytes are circled. 110× magnification, scale bars: 200 µm. (**F**) The percentage of bC27bSPs and WT larvae containing hyperplasia of visceral adipocytes (*n* = 40–50 for each groups). * *p* < 0.01, and ** *p* < 0.005.

To evaluate the importance of miR-27b in early zebrafish adipogenesis, we analyzed bC27bSPs larvae and WT siblings at the same stage fed either a LFD or HFD (Figure 6A,B). Both body weight and length were markedly increased in bC27bSPs fish that were fed either diet when compared with WT during larval development (Figure 6C,D). In addition, we compared the size of Oil Red O-stained visceral adipocytes at 24 dpf. We detected an increased mass of visceral adipocytes (Figure 6E) and an increased percentage of zebrafish larvae containing hyperplasia of visceral adipocytes in bC27bSPs as compared with WT zebrafish (Figure 6F). Thus, miR-27b depletion resulted in hyperplasia of visceral adipocytes in zebrafish juvenile, implicating an effect of miR-27b expression on post-embryonic growth and larval adipocyte formation in zebrafish.

### 2.7. Adult bC27bSPs are Plump and Have Increased White Adipose Tissue Mass

**Figure 7:**
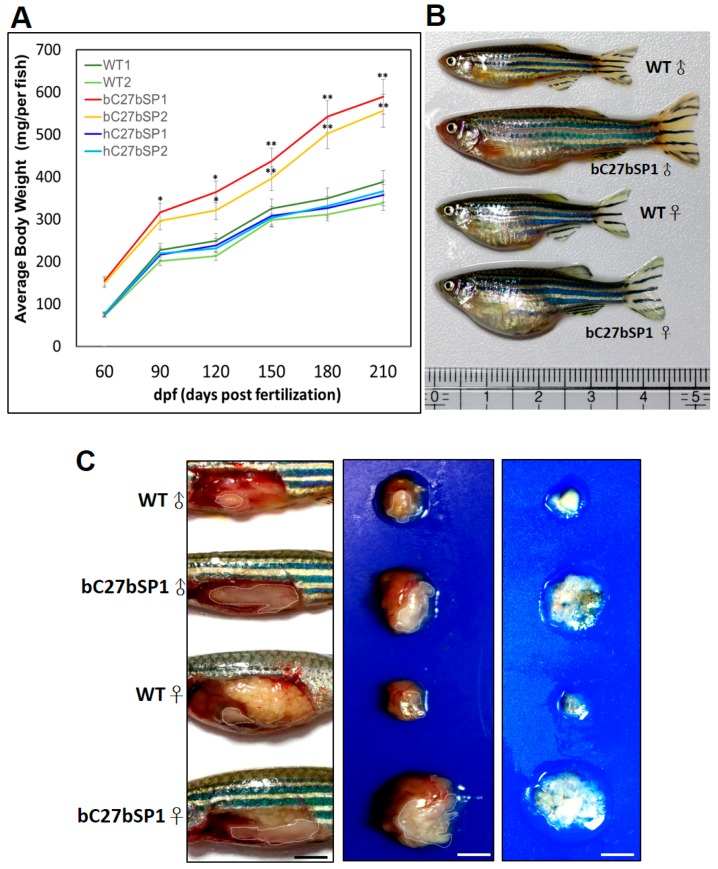

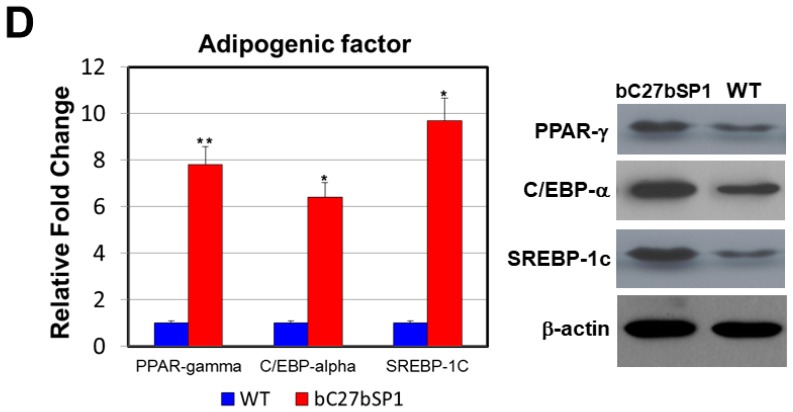
miR-27b depletion induces weight gain and increases fat mass in adult bC7aSPs. (**A**) bC27bSPs, hC27bSPs and WT were fed a HFD for 210 days. Serial body weight of each zebrafish was monitored monthly (*n* = 20–25). * *p* < 0.01, and ** *p* < 0.005. (**B**) Representative images of male and female bC27bSP1 and WT fed either a HFD for five months. (**C**) miR-27b depletion leads to greater abdominal fat (left), larger internal organs (middle) and the increased intra-abdominal fat pad size is shown (right). Adipose tissues are circled. 40× magnification, scale bars: 25 mm (**D**) Expression of adipogenic genes and proteins in abdominal fat of WT and bC27bSP1. *PPAR-γ*, *C/EBP-β*, and *SREBP-1c* mRNA (left, qRT-PCR) and proteins (right, western blot). * *p* < 0.01, and ** *p* < 0.005.

To determine whether miR-27b depletion might have functional relevance in adipose tissues, we examined the phenotype of the white adipose tissues (WAT) of adult bC27bSPs and found that they had a hypertrophic response to obesity or overweightness. Over the course of four months, we found that bC27bSPs had a significantly sharp response to being fed the HFD. Consistent with the growth curve, the body weight in bC27bSPs adults was dramatically increased over the five months (Figure 7A). bC27bSP1 adults were plump and larger than WT adults that were fed a HFD (Figure 7B). This diet-induced weight gain was accompanied by a marked increase in fat accumulation as determined by gross observation (Figure 7C, left), size of internal organs (Figure 7C, middle) and fat depot explants (Figure 7C, right). We additionally examined whether miR-27b-SP modulates hypertrophy in the WAT of bC27bSPs accompanying adipogenesis. The hypertrophic WAT was accompanied by an increase in expression of adipogenic transcriptional factors, including *PPAR-γ*, *C/EBP-α*, and *SREBP-1c* (Figure 7D). These results suggest that miR-27b depletion accelerates adipocyte differentiation in zebrafish through the upregulation of adipogenic transcriptional factors that are involved in adipogenesis.

## 3. Discussion

The microRNA miR-27b is involved in numerous metabolic processes, which are implicated in several diseases, including lipid metabolism [22,33], atherosclerosis [34,35], insulin resistance, and type-2 diabetes [36]. Despite its importance, there have been few functionally validated relevant metabolic models and in vivo studies are lacking. In this study, we identified miR-27b functions as negative regulator for both lipogenesis and adipogenesis in zebrafish. We report the novel finding that miR-27b depletion causes endotrophic and intravascular lipid accumulation and hyperlipidemia, as well as an increased expression of several lipogenic marker genes in zebrafish. Recent reports have identified miR-27b as a regulatory hub in lipid metabolism, with many confirmed metabolic targets [22,34,35,37]. Vickers et al., predicted miR-27b to be a regulatory hub in lipid metabolism [22]. They demonstrate that hepatic miR-27b is responsive to lipid levels and regulates the expression of two key metabolic genes, *angiopoietin-like 3* (*ANGPTL3*) and *glycerol-3-phosphate acyltransferase 1* (*GPAM*), which have been previously implicated in the pathobiology of dyslipidemia [38,39]. We additionally found the lipid accumulation in C27bSPs results from upregulated genes involved in de novo FFA synthesis pathways, resulting in later morbidity concomitant with different levels of hepatic steatosis. Our results suggest parallel lipid metabolic pathways for miR-27b expression in vivo and strong similarities between the lipid metabolisms of miR-27b-mediated regulation in mammals and zebrafish.

To more clearly define the physiological role of endogenous miR-27b in the hepatic response, we generated a liver-specific miR-27b-SP transgenic zebrafish model, hC27bSPs. Remarkably, hC27bSPs developed physiological NAFLD at the larval stages and NASH at the adult stage. Our results are in agreement with a recent study, showing that miR-27b inhibition restored cytoplasmic lipid droplets in rat hepatic stellate cells (HSCs) [40]. Vickers et al. reported that miR-27b was specifically found to participate in lipid metabolism in the liver, and found that miR-27b is significantly increased in HFD livers when compared to normal mouse livers [22]. They introduced miR-27b mimics or inhibitors (antagomiRs) into human hepatocytes (Huh7 cells). The overexpression of miR-27b mimics resulted in a significant increase in the expression of four lipogenic genes, *PPAR-γ*, *ANGPTL3*, *N-deacetylase/Nsulfotransferase 1* (*NDST1*), and *GPAM*, and the inhibition of endogenous miR-27b significantly upregulated the same four genes. Vacaru et al., demonstrated that zebrafish liver steatosis is associated with markers of UPR activation and robust UPR induction can cause steatosis [41]. In addition, a miR-27a/b inhibitor markedly increased the production of proinflammatory cytokines production, such as IL-1β, IL-6, MCP-1, and TNF-α [34]. Conversely, the inflammatory response was decreased by treatment of cells with a miR-27a/b mimic. Correspondingly, we provided evidence that miR-27b depletion in the liver is responsible for the development of hepatic steatosis, and even steatohepatitis, which have further combinational effects on inflammatory response and ER stress.

Several miRNAs, including miR-27, have been implicated in the process of accelerating or inhibiting preadipocyte differentiation [42,43,44,45,46]. Recently, results from three research groups have suggested that miR-27b is a negative regulator of adipocyte differentiation. Zou et al., reported that persimmon tannin inhibited adipocyte differentiation of 3T3-L1 cells through regulation of PPAR-γ, C/EBP-α, and miR-27 in the early stage of adipogenesis [19]. Karbiener et al., found that the anti-adipogenic effect of miR-27b in hMADS cells results from suppression of PPAR-γ [18]. Kang et al., demonstrated that miR-27a and b suppress adipogenesis by targeting PHB and impairing mitochondrial biogenesis and function in hMADS cells [20]. It was demonstrated that miR-27b depletion is sensitive to HFD-induced adipocyte hyperplasia, which at least partially accounts for the weight gain or obesity in bC27bSPs, showing that the bC27bSPs can be used as a model for early-onset weight gain. bC27bSPs grew fast during the larval stages (10−21 dpf), resulting in an increased body weight and length at larval stages. The increased body weight could be the result of a “head-start” in growth, caused by early-life lipid accumulation, which may be associated with impairment of lipid metabolism and an energy consumption deficient state, thereby affecting the postembryonic growth of zebrafish [47,48].

Based upon appearance and examination of adipose depots, larval bC27bSPs had modestly increased fat mass and bC27bSPs young adults had significantly increased fat mass when compared with controls. The fact that this effect substantially increased in adults indicates that bC27bSPs are sensitive to age-associated weight gain. In addition, our results suggest that the increased fat mass in bC27bSPs was caused by an increase in cell number (hyperplasia of adipocytes). Correspondingly, miR-27 was found to inhibit adipocyte differentiation, which is closely associated with the onset of obesity [19,49]. miR-27b acts as a negative regulator of adipogenesis in rat, mouse, and human cell models [18,19,20,44]. Chan et al., reported that ginsenoside-Rb1 can downregulate miR-27b activity, which in turn promotes PPAR-γ expression and adipogenesis [50]. However, miR-27b has been found to promote adipogenesis. Kong et al., identified that miR-27b is a central upstream regulator of Prdm16 to control browning of WAT [21]. “Browning” of white fat has become a current focus in the ongoing fight against obesity [51,52]. Consequently, targeting miR-27b may promote energy expenditure that is mediated by WAT conversion to brown adipose tissue (BAT), and potentially prevent obesity. Although adipose tissue has been described in adult zebrafish and its physiological and morphological similarities to that of mammals have been demonstrated [53], little is known about BAT development in zebrafish. Our in vivo observations indicate that miR-27b depletion yielded adipocyte hyperplasia by the induction of several adipogenic marker genes, including PPAR-γ, C/EBP-α, and SREBP-1c, at terminal differentiation of adipocyte in bC27bSP1. Our results suggest that the increase of adipose volume in bC27bSPs results from increased adipogenesis. We hypothesize that that miR-27b may mediate fat accumulation much better than fuel metabolism for inhibiting adipose tissue by energy expenditure. This finding highlights the potential importance of functional equivalences of miR-27b for zebrafish adipose tissue, which has also been shown to be closely related to the general development and size of zebrafish.

In conclusion, our results show that chronic depletion of miR-27b causes an increase in total lipid contents resulting the early onset of hyperlipidemia and NAFLD phenotypes in zebrafish. Chronic depletion of miR-27b leads to increased adipogenesis, which in turn, drove fat accumulation (adipocyte hyperplasia) and enhanced weight gain in zebrafish. The proposed juvenile C27bSP model showed altered pathological states, such as hepatic steatosis, and, more variably, steatohepatitis and hyperlipidemia. Aged C27bSPs with transmissible NASH-like and obesity phenotypes (different levels of severe hepatic steatosis and fat mass) are promising models for studying human metabolic diseases, such as hypertriglyceridemia, obesity, and diabetes.

## 4. Materials and Methods

### 4.1. Plasmid Construction and Oligonucleotides

To generate transgenic miR-27b cluster sponge elements (miR-27b-SP), we introduced 10 copies each of complementary sequences to miR-27b (MI0001929), each with mismatches at positions 9–12, into the 3′ UTR of mCherry in the beta-actin constructs [31]. The mutant version of miR-27b-SP (miR-27b-SP-mut) was generated with three nucleotides of the seed binding site sequence for the miR-7 binding site (ACTGTGA) were substituted (ACAAAGA). The DNA fragment carrying pri-dre-miR-27b from genomic DNA was generated by PCR using sense and antisense Pri-miR-27b (MI0001929) primers. The mature miR-27b sequences containing the miR-30e stem-loop region [54] and the miR-27b targeting sequences (TS; 5′-TGCAGAACTTAGCCACTGTGAA-3′) and its mutant sequence (mTS; 5′-TGCAGAACTTAGCCACAAAGAA-3′) were synthesized as DNA oligonucleotides (Invitrogen, Waltham, MA, USA). All DNA fragments were placed into the 3′ UTR of the pb-Act-eGFP or pb-Act-mCherry plasmids which was derivative pLF2.8 constructs [30,31,32,55]. The resulting vectors were designated as pb-Act-miR-27b, pb-Act-miR-27b-mut, pb-Act-miR-27b-SP, pb-Act-miR-27b-SP-mut, pb-Act-EGFP-miR-27b-TS, pb-Act-EGFP-miR-27b-TS-mut, pb-Act-mCherry-miR-27b-SP, pb-Act-mCherry-miR-27b-SP-mut, pb-Act-eGFP-miR-27b-TS, and pb-Act-eGFP-miR-27b-TS-mut. The sequences of all the primers and oligonucleotides used in this work are listed in Table 1. The miR-27b antisense oligonucleotide (miR-27b-ASO) and its negative control (miR-27b-ASO-NC) were synthesized by GenePharma (Shanghai, China) to perform miR-27b loss-of-function experiments. The miR-27b mimic and its negative control (miR-NC) were ordered from Ribobio (Guangzhou, China) Sigma-Aldrich (Sigma Genosys, St. Louis, MO, USA) to perform miR-27b gain-of-function experiments.

### 4.2. Generation and Feeding of hC27bSP and bC27bSP Transgenic Zebrafish

The transgenic zebrafish line hC27bSPs (Tg(-2.8fabp10a:mCherry-miR-27b-SP)) showed liver-specific expression of mCherry and miR-27b-SP (C27bSP) when driven by the L-FABP 2.8 promoter [56]. The transgenic construct pLF2.8-mCherry-miR-27b-SP is similar to constructs that we previously used to generate transgenic zebrafish lines [29,30,31,32,55,56] except that the GFP gene was replaced with the mCherry gene, and that miR-27b-SP was inserted at the 3′ end. bC27bSPs (Tg(-2.5β-Act:mCherry-miR-27b-SP)) showed global C27bSP expression when driven by the promoter of the *β-actin1* gene (GenBank Accession No. NW_001878018). For experimental assays of zebrafish larvae, zebrafish larvae were fed twice per day, starting at the 14 dpf, with either low fat diet (LFD; AZOO zebrafish LFD diet, Taikong Corp., New Taipei, Taiwan) or high fat diet (HFD; 10% crude fat added to AZOO zebrafish LFD diet) for seven days. The zebrafish were maintained in a controlled environment with a 14/10-h light/dark cycle at 28 °C. This study was approved by the Animal Ethics Committee of the National Taiwan Ocean University (permit number NTOU-104042 and 104016; permit date 24 December 2015 and 18 December 2015 respectively).

### 4.3. Cell Culture and Transfection

The zebrafish (*Danio rerio*) cell lines ZFL (CRL-2643, liver cell line) and ZEM2S (CRL-2147, fibroblast cell line derived from zebrafish embryos) and the rainbow trout (*Oncorhynchus mykiss*) cell line SOB-15 (CRL-2301, liver cell line) were obtained from the American Type Culture Collection (ATCC, Manassas, WV, USA) and maintained according to the supplier’s guidelines. Cells were propagated at 28 °C in DMEM, supplemented with 10% (*v*/*v*) fetal calf serum (FCS) and 25 µg/mL of gentamicin (Gibco BRL, Grand Island, NY, USA) in 5% CO_2_. Lipofectamine™ 2000 (Invitrogen) was used according to the manufacturer recommendations for the transient transfection studies.

### 4.4. Fluorescent Reporter Assay

For in vitro assays, ZFL, ZEM2S or SOB-15 cells were co-transfected with either pb-Act-eGFP-miR-27b-TS, pb-Act-eGFP-miR-27b-TS-mut, pb-Act-miR-27b or pb-Act-miR-27b-mut in a 48-well plate followed by transfection with pb-Act-miR-27b-SP or pb-Act-miR-27b-SP-mut. A separate RFP expression vector, pDsRed2-N1 (Clontech, Mountain View, CA, USA), was used for the normalization of expression levels. The cells were lysed 72 h later and the proteins were harvested. The eGFP and RFP fluorescence intensities were detected with a F-4500 Fluorescence Spectrophotometer (Hitachi, Tokyo, Japan). For in vivo assays, zebrafish embryos were co-microinjected with either pb-Act-eGFP-miR-27b-TS, pb-Act-eGFP-miR-27b-TS-mut, pb-Act-miR-27b or pb-Act-miR-27b-mut and pb-Act-mCherry-miR-27b-SP or pb-Act-mCherry-miR-27b-SP-mut. Antisense oligonucleotides (ASOs) were injected at a working dilution of 50 nM and miR-27b mimics were injected at a range of 0.05–0.2 ng into stage one embryos.

### 4.5. Real-Time RT-PCR and Quantification of Mature miR-27b

Total RNAs were extracted using TRIzol reagent (Invitrogen) or the RNeasy Mini Kit (Qiagen, Crawley, UK) and reverse transcribed using the first-strand cDNA synthesis kit (Thermo Fisher Scientific, Waltham, MA, USA). Real-time RT-PCR was performed using a StepOne™ Real-Time PCR System (Applied Biosystems, Foster City, CA, USA). Briefly, cDNA was amplified by 40 cycles of 95 °C for 15 s and 60 °C for 1 min using the Fast SYBR^®^ Green Master Mix (Applied Biosystems) and specific primers. The GenBank accession numbers for the selected genes and primer sequences are indicated in our previous studies [29,30,31,32]. Each sample was analyzed in triplicate. *β-actin* level was quantified as an internal control for qRT-PCR. For the quantification of mature miR-27b levels, the levels of mature miRNAs were quantified stem-loop RT primer (SRT) as described previously [36,37] and the following forward/reverse primers: dre-miR-27b SRT 5′-GTTGGCTCTGGTGCAGGGTCCGAGGTATTCGCACCAGAGCCAACTGCAGA-3′; dre-miR-27b sense 5′-GGGTTCACAGTGGCTAAG T-3′; dre-miR-27b antisense 5′-GTGCAGGGTCCGAGGT-3′. Small RNAs were extracted using the mirVana™ miRNA Isolation Kit (AM1560; Applied Biosystems) and reverse transcribed using the TaqMan MicroRNA RT Kit (4366596; Applied Biosystems). Zebrafish U6 snRNA sense 5′-TTGGTCTGATCTGGCACATATAC-3′, antisense 5′-AAAAATATGGAGCGCTTCACG-3′, antisense RT 5′-AAAAATATGGAGCGCTTCACG-3′. U6 snRNA was used as an endogenous control and fold-induction was calculated using the *C_t_* method as follows: ∆∆*C_t_* = (*C_t_*_Target_ − *C_t_*_housekeeping_) treatment − (*C_t_*_Target_ − *C_t_*_housekeeping_) non-treatment and the final quantifications were derived from the 2^−^^∆∆^*^C^*^t^ calculation.

### 4.6. Biochemical Analyses of Zebrafish Liver and Blood Lipids

To evaluate serum triglycerides and cholesterol, zebrafish blood collected from cryo-anesthetized fish was performed, as previously described [57]. Total lipids were extracted from the zebrafish liver as previously described [29,30,31,32]. Levels of free cholesterol and triglycerides (TG) in the liver were determined by colorimetric and enzymatic assays following the manufacturers’ protocols (Sigma-Aldrich, St. Louis, MO, USA). Biochemical analyses of zebrafish liver lipids were performed, as previously described [32,58]. Hepatic MDA and H_2_O_2_ levels were determined using commercial kits (Wako, Chuo-ku, and Osaka, Japan).

### 4.7. Western Blot Analysis

Western blot analysis was carried out to determine the expression level of the transgene and the targeted protein in transgenic zebrafish livers. Total hepatic protein was extracted from the isolated liver using lysis buffer (0.5% NP-40, 20 mM Tris–HCl, pH 8.0, 100 mM NaCl, 1 mM EDTA). The protein concentration was measured using the Bio-Rad protein assay kit (Bio-Rad, Hercules, CA, USA). Hepatic protein extract (20–50 µg) was separated by electrophoresis on a 12% sodium dodecyl sulfate-polyacrylamide gel and was transferred to a polyvinylidene difluoride membrane (Bio-Rad). The membrane was blocked in 5% nonfat dried milk in phosphate-buffered saline-0.05% (*v*/*v*) Tween 20 (PBST; pH 7.4) (Sigma-Aldrich) and probed with antibodies diluted ~1:500−1000 in a 0.3% solution of bovine serum albumin in PBST. PPAR-γ, C/EBP-α and SREBP-1c antibodies were purchased from Santa Cruz. Binding of the primary antibody was detected by the binding of a horseradish peroxidase-conjugated secondary antibody and development of peroxidase-catalyzed chemiluminescence (ECL kit; Amersham Biosciences, Piscataway, NJ, USA). An anti-β-actin antibody (Sigma-Aldrich) was diluted at a 1:1000 dilution in PBST was used for detecting β-actin protein level, which was used as the loading control.

### 4.8. Lipid Staining

Sectioned samples were washed three times with PBS, followed by fixation with 10% formalin in phosphate buffer for 1 h at room temperature. After fixation, the samples were again washed with PBS and were stained with a filtered Oil Red O (Sigma-Aldrich) stock solution (0.5 g Oil Red O in 100 mL isopropyl alcohol) for 15 min at room temperature. The samples were then washed twice with H_2_O for 15 min. For whole-mount Oil Red O analysis, larvae were fixed with 4% paraformaldehyde, washed with PBS, infiltrated with a graded series of propylene glycol solutions, and stained with 0.5% Oil Red O in 100% propylene glycol overnight. Stained larvae were washed with decreasing concentrations of propylene glycol, followed by several rinses with PBS, and then transferred into an 80% glycerol solution. Stained larvae were viewed on a Leica MZ16 FA stereomicroscope and images were acquired with a digital camera (AxioCam; Carl Zeiss, Sliedrecht, The Netherlands).

### 4.9. Statistical Analysis

The Student’s *t*-test was applied to evaluate statistical significance for data sets which showed normal distributions. *p*-values of ≤0.01 or ≤0.005 were considered to indicate a significant difference between groups. All data are shown as the mean ± SEM.

## Figures and Tables

**Table 1:** Primer sequences used for this study.

Gene	Accession	Forward Primer	Reverse Primer
*PPAR-α*	NM_001102567	CTGCGGGACATCTCTCAGTC	ACCGTAAACACCTGACGACG
*PPAR-γ*	DQ839547	CCTGTCCGGGAAGACCAGCG	GTGCTCGTGGAGCGGCATGT
*C/EBP-α*	NM_131885	AACGGAGCGAGCTTGACTT	AAATCATGCCCATTAGCTGC
*SREBP-1c*	NM_001105129	CAGAGGGTGGGCATGCTGGC	ACCTGGTTCTGGATGAATCG
*il1b*	NM_212844	TGGCGAACGTCATCCAAG	GGAGCACTGGGCGACGCATA
*il6*	NM_001261449	AGACCGCTGCCTGTCTAAAA	TTTGATGTCGTTCACCAGGA
*tnfa*	NM_212859	GCTTATGAGCCATGCAGTGA	TGCCCAGTCTGTCTCCTTCT
*ifn*-*γ*	NM_212864.1	TGGGCGATCAAGGAAAACGA	TTGATGCTTTAGCCTGCCGT
*nfkb2*	NM_001001840.3	AAACAAGACGCAAGGAGCCC	GCTGAAGGAAACGTCATAGGC
*NF*-*κB*	BC162402	GAAGCATTCAGGCTCGGTGA	CAGGTCTGTCGGTCCCTTTC
*atf6*	NM_001110519	CTGTGGTGAAACCTCCACCT	CATGGTGACCACAGGAGATG
*ern2*	XM_001919315	TGACGTGGTGGAAGTTGGTA	ACGGATCACATTGGGATGTT
*ire1*	XM_009306258	TGACGTGGTGGAAGTTGGTA	ACGGATCACATTGGGATGTT
*eif2ak3(perk)*	XM_005156585	TGGGCTCTGAAGAGTTCGAT	TGTGAGCCTTCTCCGTCTTT
*hspa5(bip)*	NM_213058	AAGAGGCCGAAGAGAAGGAC	AGCAGCAGAGCCTCGAAATA
*ddit3(chop)*	NM_001082825	AAGGAAAGTGCAGGAGCTGA	TCACGCTCTCCACAAGAAGA
*β-Actin*	NM_131031.1	GCCACCTTAAATGGCCTAGCA	GCCATACAGAGCAGAAGCCA

## Abbreviations

NAFLD	nonalcoholic fatty liver disease
NASH	nonalcoholic steatohepatitis
FLD	fatty liver disease
YY1	Yin Yang 1
CB1R	cannabinoid receptor 1
CHOP-10	C/EBP homologous protein 10
PPAR-γ	peroxisome proliferator-activated receptor γ
C/EBP-α	CCAAT enhancer binding protein-α
C/EBP-β	CCAAT enhancer binding protein-β
ANGPTL3	angiopoietin-like 3
GPAM	glycerol-3-phosphate acyltransferase 1
SREBP-1c	sterol regulatory element binding transcription factor 1c
miRs	microRNAs
miR-SP	microRNA-sponge
WAT	white adipose tissue

## References

[1] Shen L., Zhang Y., Du J., Chen L., Luo J., Li X., Li M., Tang G., Zhang S., Zhu L. (2016). MicroRNA-23a regulates 3T3-L1 adipocyte differentiation. Gene.

[2] Song G., Xu G., Ji C., Shi C., Shen Y., Chen L., Zhu L., Yang L., Zhao Y., Guo X. (2014). The role of microRNA-26b in human adipocyte differentiation and proliferation. Gene.

[3] Lie S., Morrison J.L., Williams-Wyss O., Suter C.M., Humphreys D.T., Ozanne S.E., Zhang S., MacLaughlin S.M., Kleemann D.O., Walker S.K. (2016). Impact of maternal undernutrition around the time of conception on factors regulating hepatic lipid metabolism and microRNAs in singleton and twin fetuses. Am. J. Physiol. Endocrinol. Metab..

[4] Massart J., Katayama M., Krook A. (2016). microManaging glucose and lipid metabolism in skeletal muscle: Role of microRNAs. Biochim. Biophys. Acta.

[5] Baffy G. (2015). MicroRNAs in Nonalcoholic Fatty Liver Disease. J. Clin. Med..

[6] Tessitore A., Cicciarelli G., del Vecchio F., Gaggiano A., Verzella D., Fischietti M., Mastroiaco V., Vetuschi A., Sferra R., Barnabei R. (2016). MicroRNA expression analysis in high fat diet-induced NAFLD-NASH-HCC progression: Study on C57BL/6J mice. BMC Cancer.

[7] Parrizas M., Novials A. (2016). Circulating microRNAs as biomarkers for metabolic disease. Best Pract. Res. Clin. Endocrinol. Metab..

[8] Willeit P., Skroblin P., Kiechl S., Fernandez-Hernando C., Mayr M. (2016). Liver microRNAs: Potential mediators and biomarkers for metabolic and cardiovascular disease?. Eur. Heart J..

[9] Rocic P. (2016). Can microRNAs be biomarkers or targets for therapy of ischemic coronary artery disease in metabolic syndrome?. Curr. Drug Targets.

[10] Deiuliis J.A. (2016). MicroRNAs as regulators of metabolic disease: Pathophysiologic significance and emerging role as biomarkers and therapeutics. Int. J. Obes. (Lond.).

[11] Ferreira D.M., Simao A.L., Rodrigues C.M., Castro R.E. (2014). Revisiting the metabolic syndrome and paving the way for microRNAs in non-alcoholic fatty liver disease. FEBS J..

[12] Fernandez-Hernando C., Ramirez C.M., Goedeke L., Suarez Y. (2013). MicroRNAs in metabolic disease. Arterioscler. Thromb. Vasc. Biol..

[13] Zampetaki A., Mayr M. (2012). MicroRNAs in vascular and metabolic disease. Circ. Res..

[14] Otsuka M., Kishikawa T., Yoshikawa T., Yamagami M., Ohno M., Takata A., Shibata C., Ishibashi R., Koike K. (2017). MicroRNAs and liver disease. J. Hum. Genet..

[15] Sobolewski C., Calo N., Portius D., Foti M. (2015). MicroRNAs in fatty liver disease. Semin. Liver Dis..

[16] Szabo G., Bala S. (2013). MicroRNAs in liver disease. Nat. Rev. Gastroenterol. Hepatol..

[17] Wang X.W., Heegaard N.H., Orum H. (2012). MicroRNAs in liver disease. Gastroenterology.

[18] Karbiener M., Fischer C., Nowitsch S., Opriessnig P., Papak C., Ailhaud G., Dani C., Amri E.-Z., Scheideler M. (2009). microRNA miR-27b impairs human adipocyte differentiation and targets PPAR-γ. Biochem. Biophys. Res. Commun..

[19] Zou B., Ge Z., Zhu W., Xu Z., Li C. (2015). Persimmon tannin represses 3T3-L1 preadipocyte differentiation via up-regulating expression of miR-27 and down-regulating expression of peroxisome proliferator-activated receptor-γ in the early phase of adipogenesis. Eur. J. Nutr..

[20] Kang T., Lu W., Xu W., Anderson L., Bacanamwo M., Thompson W., Chen Y.E., Liu D. (2013). MicroRNA-27 (miR-27) targets prohibitin and impairs adipocyte differentiation and mitochondrial function in human adipose-derived stem cells. J. Biol. Chem..

[21] Kong X., Yu J., Bi J., Qi H., Di W., Wu L., Wang L., Zha J., Lv S., Zhang F. (2015). Glucocorticoids transcriptionally regulate miR-27b expression promoting body fat accumulation via suppressing the browning of white adipose tissue. Diabetes.

[22] Vickers K.C., Shoucri B.M., Levin M.G., Wu H., Pearson D.S., Osei-Hwedieh D., Collins F.S., Remaley A.T., Sethupathy P. (2013). MicroRNA-27b is a regulatory hub in lipid metabolism and is altered in dyslipidemia. Hepatology.

[23] Zeituni E.M., Farber S.A. (2016). Studying Lipid Metabolism and Transport During Zebrafish Development. Methods Mol. Biol..

[24] Gao Y., Dai Z., Shi C., Zhai G., Jin X., He J., Lou Q., Yin Z. (2016). Depletion of myostatin b promotes somatic growth and lipid metabolism in zebrafish. Front. Endocrinol..

[25] Cui Y., Lv S., Liu J., Nie S., Chen J., Dong Q., Huang C., Yang D. (2016). Chronic perfluorooctanesulfonic acid exposure disrupts lipid metabolism in zebrafish. Hum. Exp. Toxicol..

[26] Ho J.C., Hsiao C.D., Kawakami K., Tse W.K. (2016). Triclosan (TCS) exposure impairs lipid metabolism in zebrafish embryos. Aquat. Toxicol..

[27] Anderson J.L., Carten J.D., Farber S.A. (2011). Zebrafish lipid metabolism: From mediating early patterning to the metabolism of dietary fat and cholesterol. Methods Cell Biol..

[28] Schlegel A. (2012). Studying non-alcoholic fatty liver disease with zebrafish: A confluence of optics, genetics, and physiology. Cell. Mol. Life Sci..

[29] Shieh Y.S., Chang Y.S., Hong J.R., Chen L.J., Jou L.K., Hsu C.C., Her G.M. (2010). Increase of hepatic fat accumulation by liver specific expression of Hepatitis B virus X protein in zebrafish. Biochim. Biophys. Acta.

[30] Her G.M., Hsu C.C., Hong J.R., Lai C.Y., Hsu M.C., Pang H.W., Chan S.K., Pai W.Y. (2011). Overexpression of gankyrin induces liver steatosis in zebrafish (*Danio rerio*). Biochim. Biophys. Acta.

[31] Her G.M., Pai W.Y., Lai C.Y., Hsieh Y.W., Pang H.W. (2013). Ubiquitous transcription factor YY1 promotes zebrafish liver steatosis and lipotoxicity by inhibiting CHOP-10 expression. Biochim. Biophys. Acta.

[32] Pai W.Y., Hsu C.C., Lai C.Y., Chang T.Z., Tsai Y.L., Her G.M. (2013). Cannabinoid receptor 1 promotes hepatic lipid accumulation and lipotoxicity through the induction of SREBP-1c expression in zebrafish. Transgen. Res..

[33] Goedeke L., Rotllan N., Ramirez C.M., Aranda J.F., Canfran-Duque A., Araldi E., Fernandez-Hernando A., Langhi C., de Cabo R., Baldan A. (2015). miR-27b inhibits LDLR and ABCA1 expression but does not influence plasma and hepatic lipid levels in mice. Atherosclerosis.

[34] Xie W., Li L., Zhang M., Cheng H.P., Gong D., Lv Y.C., Yao F., He P.P., Ouyang X.P., Lan G. (2016). MicroRNA-27 prevents atherosclerosis by suppressing lipoprotein lipase-induced lipid accumulation and inflammatory response in apolipoprotein E knockout mice. PLoS ONE.

[35] Chen W.J., Yin K., Zhao G.J., Fu Y.C., Tang C.K. (2012). The magic and mystery of microRNA-27 in atherosclerosis. Atherosclerosis.

[36] Wang J.M., Tao J., Chen D.D., Cai J.J., Irani K., Wang Q., Yuan H., Chen A.F. (2014). MicroRNA miR-27b rescues bone marrow-derived angiogenic cell function and accelerates wound healing in type 2 diabetes mellitus. Arterioscler. Thromb. Vasc. Biol..

[37] Zhang M., Wu J.F., Chen W.J., Tang S.L., Mo Z.C., Tang Y.Y., Li Y., Wang J.L., Liu X.Y., Peng J. (2014). microRNA-27a/b regulates cellular cholesterol efflux, influx and esterification/hydrolysis in THP-1 macrophages. Atherosclerosis.

[38] Linden D., William-Olsson L., Ahnmark A., Ekroos K., Hallberg C., Sjogren H.P., Becker B., Svensson L., Clapham J.C., Oscarsson J. (2006). Liver-directed overexpression of mitochondrial glycerol-3-phosphate acyltransferase results in hepatic steatosis, increased triacylglycerol secretion and reduced fatty acid oxidation. FASEB J..

[39] Musunuru K., Pirruccello J.P., Do R., Peloso G.M., Guiducci C., Sougnez C., Garimella K.V., Fisher S., Abreu J., Barry A.J. (2010). Exome sequencing, ANGPTL3 mutations, and familial combined hypolipidemia. N. Engl. J. Med..

[40] Ji J., Zhang J., Huang G., Qian J., Wang X., Mei S. (2009). Over-expressed microRNA-27a and 27b influence fat accumulation and cell proliferation during rat hepatic stellate cell activation. FEBS Lett..

[41] Vacaru A.M., di Narzo A.F., Howarth D.L., Tsedensodnom O., Imrie D., Cinaroglu A., Amin S., Hao K., Sadler K.C. (2014). Molecularly defined unfolded protein response subclasses have distinct correlations with fatty liver disease in zebrafish. Dis. Model. Mech..

[42] Xie H., Lim B., Lodish H.F. (2009). microRNAs induced during adipogenesis that accelerate fat cell development are downregulated in obesity. Diabetes.

[43] Wang Q., Li Y.C., Wang J., Kong J., Qi Y., Quigg R.J., Li X. (2008). miR-17-92 cluster accelerates adipocyte differentiation by negatively regulating tumor-suppressor Rb2/p130. Proc. Natl. Acad. Sci. USA.

[44] Lin Q., Gao Z., Alarcon R.M., Ye J., Yun Z. (2009). A role of miR-27 in the regulation of adipogenesis. FEBS J..

[45] Hilton C., Neville M., Karpe F. (2013). microRNAs in adipose tissue: Their role in adipogenesis and obesity. Int. J. Obes..

[46] Peng Y., Yu S., Li H., Xiang H., Peng J., Jiang S. (2014). microRNAs: Emerging roles in adipogenesis and obesity. Cell Signal..

[47] Flynn E.J., Trent C.M., Rawls J.F. (2009). Ontogeny and nutritional control of adipogenesis in zebrafish (*Danio rerio*). J. Lipid Res..

[48] Den Broeder M.J., Kopylova V.A., Kamminga L.M., Legler J. (2015). Zebrafish as a model to study the role of peroxisome proliferating-activated receptors in adipogenesis and obesity. PPAR Res..

[49] Sun L., Trajkovski M. (2014). miR-27 orchestrates the transcriptional regulation of brown adipogenesis. Metabolism.

[50] Chan L.S., Yue P.Y., Kok T.W., Keung M.H., Mak N.K., Wong R.N. (2012). Ginsenoside-Rb1 promotes adipogenesis through regulation of PPAR-γ and microRNA-27b. Horm. Metab. Res..

[51] Seale P., Conroe H.M., Estall J., Kajimura S., Frontini A., Ishibashi J., Cohen P., Cinti S., Spiegelman B.M. (2011). Prdm16 determines the thermogenic program of subcutaneous white adipose tissue in mice. J. Clin. Investig..

[52] May F.J., Baer L.A., Lehnig A.C., So K., Chen E.Y., Gao F., Narain N.R., Gushchina L., Rose A., Doseff A.I. (2017). Lipidomic adaptations in white and brown adipose tissue in response to exercise demonstrate molecular species-specific remodeling. Cell Rep..

[53] Imrie D., Sadler K.C. (2010). White adipose tissue development in zebrafish is regulated by both developmental time and fish size. Dev. Dyn..

[54] Dong M., Fu Y.F., Du T.T., Jing C.B., Fu C.T., Chen Y., Jin Y., Deng M., Liu T.X. (2009). Heritable and lineage-specific gene knockdown in zebrafish embryo. PLoS ONE.

[55] Chen L.J., Hsu C.C., Hong J.R., Jou L.K., Tseng H.C., Wu J.L., Liou Y.C., Her G.M. (2008). Liver-specific expression of p53-negative regulator mdm2 leads to growth retardation and fragile liver in zebrafish. Dev. Dyn..

[56] Her G.M., Chiang C.C., Chen W.Y., Wu J.L. (2003). In vivo studies of liver-type fatty acid binding protein (L-FABP) gene expression in liver of transgenic zebrafish (*Danio rerio*). FEBS Lett..

[57] Pedroso G.L., Hammes T.O., Escobar T.D., Fracasso L.B., Forgiarini L.F., da Silveira T.R. (2012). Blood collection for biochemical analysis in adult zebrafish. J. Vis. Exp..

[58] Yeh K.Y., Lai C.Y., Lin C.Y., Hsu C.C., Lo C.P., Her G.M. (2017). ATF4 overexpression induces early onset of hyperlipidaemia and hepatic steatosis and enhances adipogenesis in zebrafish. Sci. Rep..

